# High Expression of RAB32 Predicts Adverse Outcomes: A Potential Therapeutic Target for Glioblastoma

**DOI:** 10.7150/jca.96162

**Published:** 2024-10-28

**Authors:** Liji Huang, Yue Chi, Xiangyue Su, Hongyu Zhang, Yanfei Cao, Xindi Wang, Sinan Zhang, Xudong Jiang, Lina Zhang

**Affiliations:** 1 Departments of Laboratory Diagnosis, Liuzhou Traditional Chinese Medical Hospital, Liuzhou, China.; 2 Departments of Laboratory Diagnosis, Daqing Oilfield General Hospital, Daqing, China.; 3 Department of Laboratory Medicine, Key Laboratory of Precision Medicine for Viral Diseases, Guangxi Health Commission Key Laboratory of Clinical Biotechnology, Liuzhou People's Hospital, Liu Zhou, China.; 4 Harbin Medical University (Daqing), Daqing, China.

**Keywords:** Glioblastoma, RAB32, Methylation, Immune infiltration, Bioinformatics, Prognosis

## Abstract

RAB32 is a potential prognostic marker that is overexpressed in a variety of cancers. The purpose of this study was to investigate the expression and function of RAB32 in glioblastoma (GBM). The RAB32 expression data were obtained by accessing the TCGA, CGGA and GEPIA databases and were verified by western blot and immunohistochemistry. The prognostic value of RAB32 methylation was carefully examined using cBioPortal and MethSurv. GSEA was used to analyze cancer-related signaling pathways that may be activated by high RAB32 expression. The correlation between RAB32 and GBM infiltration was studied by accessing the TISIDB database. The effects of RAB32 on the proliferation, migration and invasion of GBM cells were predicted by colony formation assay, CCK-8 assay and Transwell assay. In this study, RAB32 expression was upregulated in GBM compared to normal brain tissue. Survival analysis showed that high expression of RAB32 was an independent risk factor for overall survival in glioma patients. RAB32 methylation was negatively correlated with RAB32 expression, and the overall survival rate of patients with RAB32 hypomethylation was lower than that of patients with RAB32 hypermethylation. Through functional enrichment analysis, we found that RAB32 overexpression significantly activated multiple signaling pathways. The immunoassay results showed that RAB32 expression was correlated with immune infiltration of the tumor microenvironment. Knocking down the expression of the RAB32 gene significantly inhibited the proliferation, migration and invasion of glioma cells. Our results show that RAB32 is a key factor affecting the prognosis of patients with GBM, and its targeting may provide a new treatment for patients with GBM.

## Introduction

Glioblastoma (GBM) constitutes a substantial proportion of primary brain tumors, accounting for nearly 30% of such cases and an overwhelming 80% of malignant brain tumors. The World Health Organization (WHO) classifies gliomas into four grades based on their histopathological characteristics: Grades I and II are designated as low-grade glioblastomas (LGG), Grade III is classified as anaplastic, and Grade IV is categorized as glioblastoma, reflecting varying degrees of malignancy^1^. GBM, in particular, not only ranks among the most lethal malignancies but also stands out as the most prevalent and invasive primary brain tumor. A major challenge in treating gliomas lies in overcoming drug resistance encountered with conventional radiotherapy and chemotherapy. Recent advancements in genomics and epigenetics have significantly enhanced prognostic capabilities for patients with glioma^2^,^3^. The molecular subtyping of gliomas has proven beneficial in facilitating timely molecular diagnosis and enabling the selection of treatment modalities tailored to clinical requirements^4^. Despite significant advancements in medical and surgical interventions, including prompt implementation of standard treatment protocols involving surgery, radiation therapy, and medication following diagnosis, the median survival rate for glioma patients remains tragically below two years, with an average survival duration of 8 months^5^. Despite the availability of numerous treatment options for gliomas, there has been limited improvement in prognosis due to challenges associated with surgical resection, rapid disease progression, and a high recurrence rate.

The RAS-related protein family is a crucial regulatory small molecule GTP-binding protein that facilitates intracellular substance transport and plays a pivotal role in cell membrane transport pathways^6^. Functionally, depending on its GTP binding state, RAB32 can modulate the function of intracellular and extracellular signaling and maintain cytoskeletal integrity^7^. Additionally, RAB32 can interact with mitochondria to induce morphological changes; however, mitochondrial dysfunction is believed to impact reactive oxygen species (ROS)-mediated energy metabolism, oxidative stress, DNA damage, and cellular apoptosis. Alterations in these factors have emerged as potential key contributors to tumorigenesis^6^-^8^. Furthermore, RAB32 participates in liver cell metabolism and autophagy processes^9^,^10^. Consequently, aberrant functioning of RAB32 may contribute to metabolic disorders and cancer development. Notably, RAB32 can activate mTORC1 signal transduction pathway leading to autophagy regulation while promoting proliferation and vitality of Hep3B and HeLa cells^11^.

Despite a substantial body of research highlighting the significance of RAB32 in tumorigenesis, the precise biological functions and molecular mechanisms underlying its role in gliomas remain elusive. This study represents the first comprehensive exploration into the biological functions of RAB32 in gliomas and its correlation with patient prognosis. Our findings provide compelling evidence that RAB32 plays a pivotal role in glioma development, thus identifying it as a novel therapeutic target for this aggressive cancer. Moreover, our study opens up avenues for enhancing patient survival rates and prognosis, offering promising prospects for improved clinical management of glioma patients.

## Materials and Methods

### Data collection

The expression of RAB32 in various tumors was analyzed using the GEPIA data online platform (http://gepia.cancer-pku.cn/)^12^ and the Tumor Immune Estimation Resource (TIMER2.0, http://timer.comp-genomics.org/)^13^. We used mainly this database for mutual verification and discussion of RAB32 expression in pan carcinoma to improve the reliability of the analysis. Glioma tissue sequencing (RNA-seq) data and clinical information data were downloaded from The Cancer Genome Atlas (TCGA, https://portal.gdc.cancer.gov)^14^ and CGGA databases (http://www.cgga.org.cn/)^15^ to generate a gene expression matrix. Correlation analysis was performed after deletion of cases with unknown or incomplete clinicopathological characteristics and the absence of prognostic follow-up data.

### Glioma and normal tissue samples

For the purpose of this study, a central nervous system (CNS) tissue microarray was acquired from Zhongke Guanghua Intelligent Biotechnology Co., Ltd. (https://www.bioaitech.com/). The microarray assembly comprises total of 109 individual samples, which include a diverse array of pathological conditions: 7 samples of WHO grade I astrocytoma, 32 samples of WHO grade II astrocytoma, 23 samples of WHO grade III anaplastic astrocytoma, 36 samples glioblastoma (GBM), and 11 samples of normal CNS tissue. It is important to note that this study did not involve the recruitment of participants or require active patient involvement. The samples utilized were sourced from Zhongke Guanghua Intelligent Biotechnology Co., Ltd., located in Xi'an, China, and were obtained in a manner that respected privacy and ethical considerations. Inclusion and Exclusion sample criteria: Glioma patients voluntarily underwent surgical treatment, and no other form of treatment was received before surgery. The postoperative pathological diagnosis was adult brain glioma. The patient had no other organ or systemic tumors or systemic diseases.

### Cell culture

The GBM cell Lines U251, U87, and A172 were purchased from the National Collection of Authenticated Cell Culture (https://www.cellbank.org.cn/) and cultured in DMEM (HyClone) supplemented with 10% fetal bovine serum. The normal astrocyte cell line SVG p12 was purchased from American Type Culture Collection (ATCC, https://www.atcc.org/) and cultured in EMEM (ATCC) supplemented with 10% fetal bovine serum. The culture media of all cell lines were supplemented with penicillin-streptomycin, and mycoplasma was detected regularly. All cell lines were grown in 5% CO2-95% O2 at 37 °C and cultured for more than 6 months after receipt.

### Immunohistochemistry

First, the tissue microarray was dewaxed, rehydrated, and incubated in a peroxidase blocker to block endogenous peroxidase activity in the sample. The microarray was then heated with sodium citrate buffer for 20 minutes to repair the antigens. The nonspecific response of the samples was blocked with goat serum and incubated with the primary antibody RAB32 (Abcam, rabbit polyclonal, 1:200) overnight at 4 °C. After the microarray was rewarmed at 37 °C for 45 min, the biotin-avidin system was used for detection. The microarrays were stained with hematoxylin and differentiated with hydrochloric acid and alcohol. Finally, the sections were dehydrated, dried, sealed with a neutral resin, and photographed with a microscope. Immunohistochemical staining intensity was evaluated by ImageJ software.

### Western blotting

RIPA buffer was used to extract the protein, and the protein concentration was detected by BCA protein analysis according to the instructions. After gel electrophoresis, the proteins were transferred to nitrocellulose membranes and incubated with 5% skim milk for 1 h at room temperature in a sealed container. The primary anti-RAB32 antibody (1:500, 25 kDa) and GAPDH (1:2000, 36 kDa) were incubated overnight at 4 °C. The secondary antibody was labeled with horseradish peroxidase, and the protein bands were visualized using ECL. The image was captured using iBright 1500 (Thermo Fisher Scientific) and detected in grayscale using ImageJ software.

### DNA methylation and genetic change analysis

RARB32 change frequency, mutation type, copy number change (CNA), and RAB32 DNA methylation data were investigated in cBioPortal (http://cbioportal.org/) from various cancers in TCGA^16^. The Kruskal‒Wallis test was used to compare RAB32 mRNA expression levels between the RAB32 CNV group and the RAB32 CNV-free group, and the Pearson correlation coefficient was used to analyze the correlation between RAB32 DNA methylation and RAB32 mRNA expression. The SMART online tool (http://www.bioinfo-zs.com/smartapp/) was used to verify the correlation between RAB32 DNA methylation levels and RAB32 mRNA expression. MethSurv online tools (https://biit.cs.ut.ee/methsurv/) were used to explore the prognostic value of RAB32 DNA methylation levels in patients with glioma. RAB32 DNA methylation data of glioma tissue samples were downloaded from CGGA (methyl_159) to evaluate the methylation levels and the prognostic value of gliomas of different grades.

### Gene Set Enrichment Analysis (GSEA)

Gene set enrichment analysis (GSEA) is a method for analyzing the microarray data of the whole-genome expression profile^17^. By analyzing gene expression profile data, we could understand how well genes are expressed in specific functional gene sets and whether they were statistically significant. After downloading CGGA RNA-seq and TCGA RNA-seq data from CGGA and TCGA, we divided the data into a high-expression group and a low-expression group according to the RAB32 expression level. In this analysis, H. avir7.2. symbols were used as the reference gene set, and GSEA 4.0.jar software was used for enrichment analysis. When the false discovery rate (FDR) Q was < 0.25, the p adjust value was < 0.05 and the normalized enrichment score (|NES |) was > 1.5, we considered the enrichment to be significant.

### Immunoinfiltration analysis

The TISIDB database (http://cis.hku.hk/TISIDB/index.php) from various public medical databases^18^, through high-throughput screening data to detect tumors, is associated with 28 immune cells. The correlation between the RAB32 mRNA expression level and tumor immune cells was detected by TISIDB data, and the ESTIMATE algorithm was used to estimate tumor purity. SIGLEC15, TIGIT, CD274, HAVCR2, PDCD1, CTLA4, LAG3 and PDCD1LG2 were the genes associated with immune checkpoints. The expression values of these 8 genes were extracted to observe the expression of the genes associated with immune checkpoints. According to the median RAB32 expression values of 393 HGG samples and 216 LGG samples obtained from the TCGA database, they were divided into high or low RAB32 expression groups. The correlation between the immune checkpoint and RAB32 was analyzed, and the expression in the high or low RAB32 expression groups was further analyzed.

### Cell transfection

U87 cells were seeded in six-well plates and allowed to reach 40% confluence. Two small interfering RNAs (siRNAs) targeting human RAB32, along with a negative control small interfering RNA (NC) lentivirus containing a non-targeting sequence, were synthesized by Suzhou Jima Gene Biotechnology Co., Ltd. The siRNAs were then transfected into U87 cells. Subsequent experiments were conducted 48 hours post-transfection to evaluate the effects of the knockdown on RAB32 expression and downstream pathways. This experimental design allowed for the investigation of the specific role of RAB32 in U87 cells and its potential implications in cellular functions.

### Colony formation assay

U87 cells were uniformly inoculated into six-well plates at a cell density of 300 cells per well. The cells were transfected with NC, siRAB32-1 or siRAB32-2 and cultured in a humid incubator with 5% CO2 at 37 °C for 3 weeks. Cells were fixed with 4% paraformaldehyde (Beijing Boaotoda Technology Co., LTD.) for 15 min and stained with 0.1% crystal violet (Biosharp) for 20 min. Using a high-resolution mobile phone, the colonies were photographed and counted under a microscope.

### CCK-8 assay

Cell Counting Kit 8 was used to explore cell proliferation. Transfected U87 cells were obtained to create a cell suspension. A total of 3000 cells/well were inoculated into 96-well plates for 24, 48, 72, 96, and 120 h. Then, 10 μl/well CCK-8 solution was added and incubated for 2 h, and the OD values of each sample were read on a 450-nm spectrophotometer. The experiment was repeated with five wells.

### Transwell assays

Transwell chambers were used (Corning, Inc., Corning, NY, USA). Cell migration and invasion were evaluated. Transfected U87 cells (2 × 104) were placed in the upper chamber and mixed evenly with serum-free medium. The lower chamber was fitted with cell-free medium containing 10% fetal bovine serum. After 48 h of incubation at 37 °C and 5% CO2, the cells were fixed with 4% paraformaldehyde and stained with 0.1% crystal violet. Three wells were set up and analyzed using ImageJ software.

### Statistical analysis

Univariate and multivariate Cox regression were used to construct a Cox proportional risk model, and Kaplan‒Meier survival analysis was conducted to analyze the relationship between RAB32 expression and overall survival time. The t-test and Wilcoxon test were used to compare the two data, and one-way analysis of variance (ANOVA) was used for data greater than or equal to the three groups. Finally, the Pearson test or Spearman test was used to evaluate the correlations between two variables. GraphPad Prism 6.0 software and SPSS 26.0 software were used for plotting and statistical analysis. All of the above statistical tests were considered significant when p < 0.05.

## Results

### RAB32 is highly expressed in many kinds of cancers

**Figure 1:**
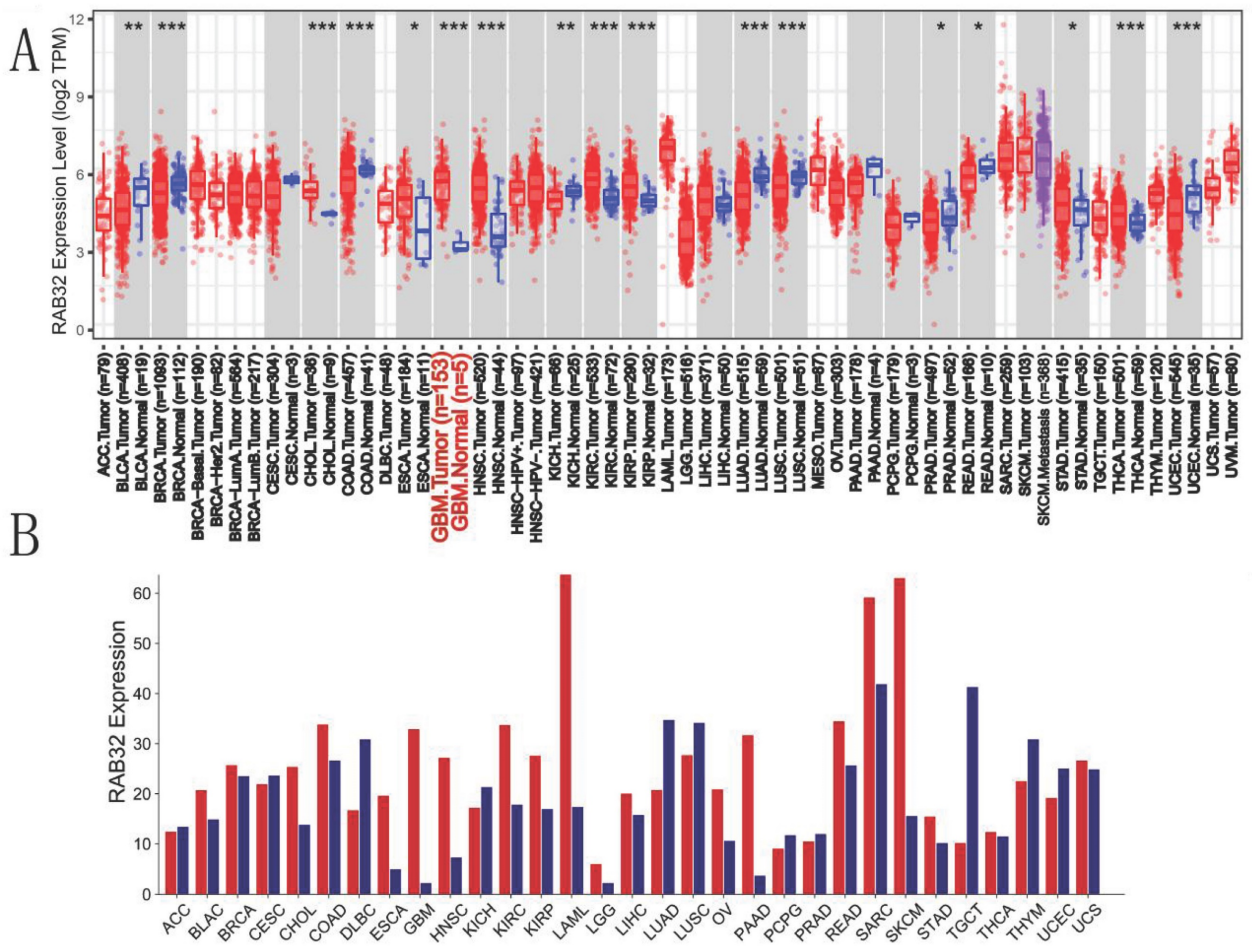
**Expression of RAB32 mRNA in various cancers.** (A) Expression of RAB32 mRNA in cancer and homologous normal tissues from the TIMER2.0 database. (B) RAB32 mRNA expression data for cancer and homologous normal tissues were obtained from the GEPIA database. *p < 0.05, **p < 0.01, and ***p < 0.001.

Our analysis of RAB32 expression across various cancers and their corresponding normal tissues, utilizing the TIMER2.0 database, revealed a notable increase in RAB32 expression levels in diverse types of cancers such as cholangiocarcinoma (CHOL), GBM, and renal clear cell carcinoma (KIRC) **(Figure 1A)**. Furthermore, to validate RAB32 expression patterns in different cancer types, we utilized the GEPIA online platform to assess RAB32 expression in tumor tissues. The findings showcased the significance of RAB32 in multiple cancer types, including but not limited to bladder urothelial carcinoma (BLCA), acute myeloid leukemia (LAML), LGG, and pancreatic adenocarcinoma (PAAD) **(Figure 1B)**. Collectively, data from both databases indicated a general upregulation of RAB32 expression in various cancers, including CHOL, ESCA, GBM, HNSC, KIRC, KIRP, and STAD.

### Overexpression of RAB32 in GBM

**Figure 2:**
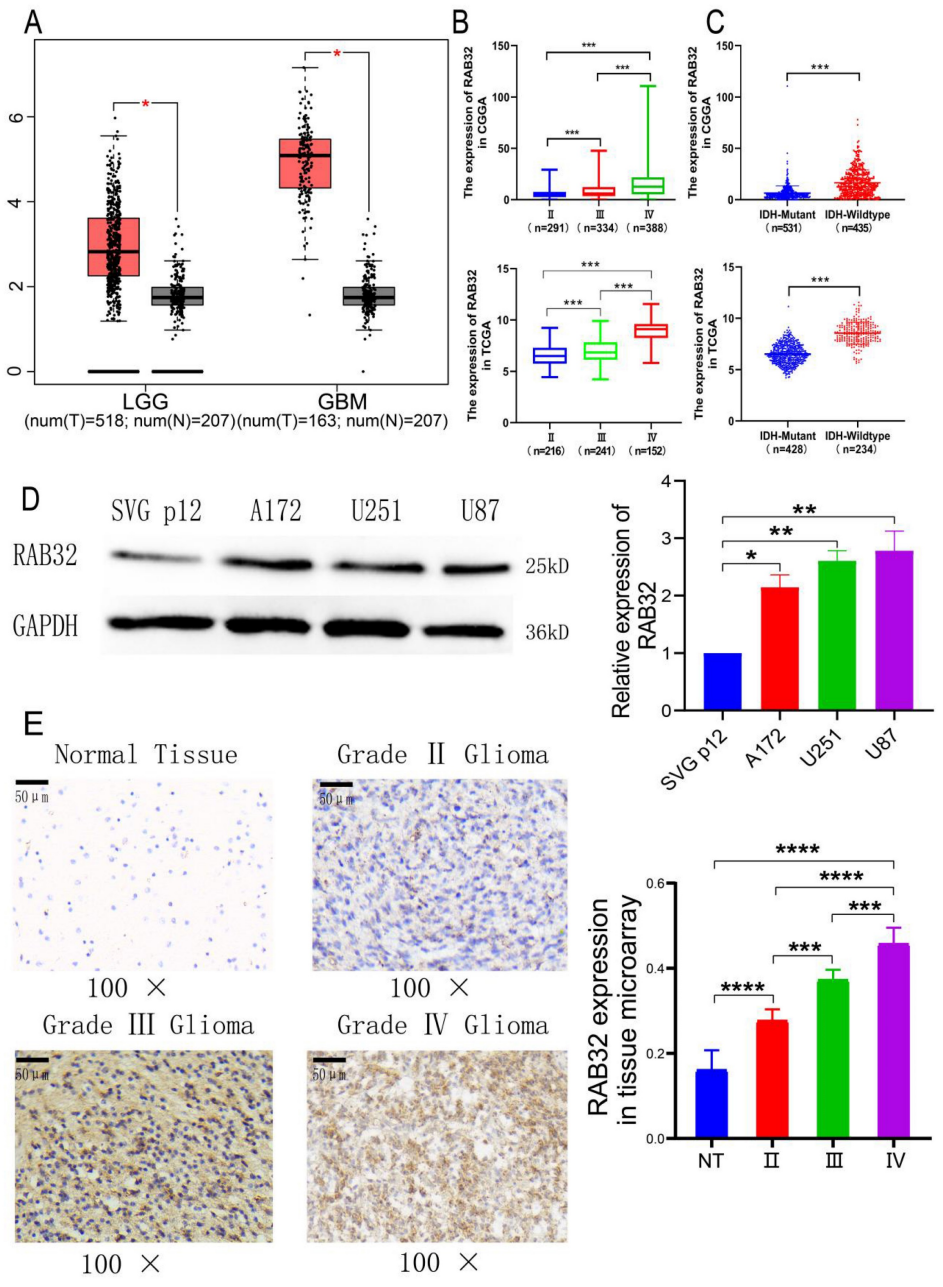
** Expression of RAB32 in glioma.** (A) RAB32 expression level between tumor and normal tissue in the GEPIA dataset. (B) RAB32 expression with glioma grade. (C) RAB32 expression with glioma IDH1 status. The above data were from CGGA, and the following data were from TCGA. (D) RAB32 protein expression in Glioblastoma cells. (E) RAB32 protein expression in the glioma microarray. *p < 0.05, **p < 0.01, ***p < 0.001.

The mRNA and protein levels of RAB32 were examined in glioma and normal brain tissues using multiple databases and western blotting assays. GEPIA analysis highlighted that RAB32 mRNA expression levels were substantially upregulated in both GBM and LGG as compared to those in normal brain tissue, with the upregulation being more pronounced in GBM **(Figure 2A)**. We next analyzed RAB32 expression in gliomas of varying grades and grouped gliomas based on diverse molecular pathological indices using the CGGA mRNA seq_693 dataset and TCGA mRNA seq_702 dataset. These findings revealed that the mRNA expression levels of RAB32 demonstrated a gradual increase in WHO II, WHO III, and WHO IV gliomas **(Figure 2B)**, and were positively correlated with the glioma grading as confirmed by high-throughput microarray analysis, which was consistent with data obtained from other sources **(Figure 2E)**. Furthermore, RAB32 mRNA expression levels exhibited positive correlations with 1p/19q codeletion and patient age, but no association with gender **(Figure S1)**. Notably, the expression of RAB32 mRNA was also higher in the IDH1 wild-type group than in the IDH1 mutant group across both databases **(Figure 2C)**. Finally, western blotting was employed to assess RAB32 expression in GBM and normal astrocyte cell lines, which revealed that RAB32 was significantly overexpressed in GBM cell lines as compared to normal astrocyte lines **(Figure 2D)**. Collectively, these results suggest that RAB32 expression plays a critical role in the malignant development of glioma, possibly underscoring its association with poor prognosis.

### RAB32 overexpression is associated with poor prognosis of glioma

**Figure 3:**
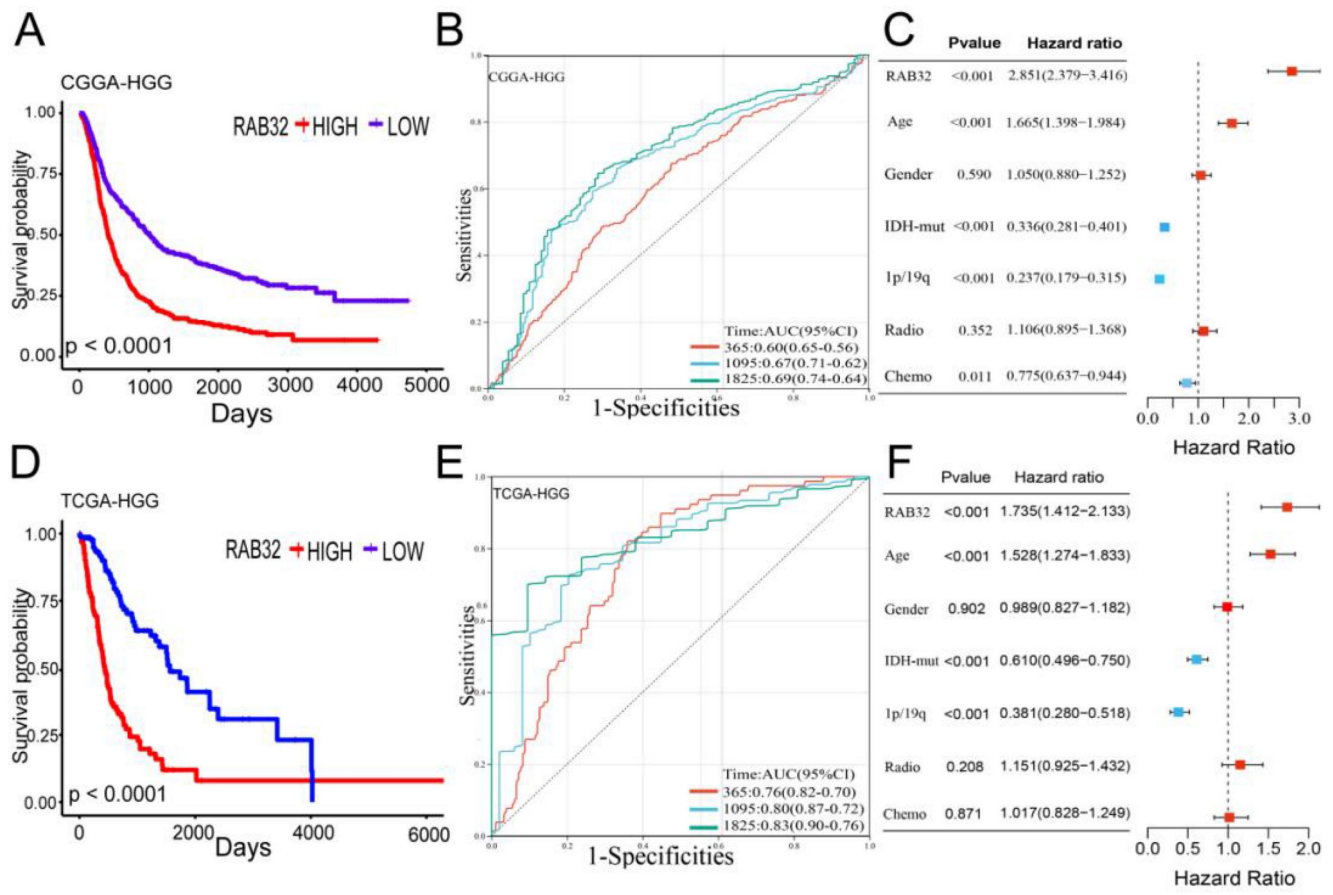
**RAB32 overexpression is associated with a poor prognosis for glioma.** (A, D) Overall survival curve based on CGGA and TCGA data. (B, E) ROC curve based on CGGA and TCGA data. (C, F) Forest map based on univariate and multivariate Cox analyses of the CGGA database.

To further verify our inferences, we used clinical prognostic data from the CGGA and TCGA databases for a survival analysis. The results of the survival analysis showed that RAB32 expression in HGG was negatively correlated with glioma survival, the overall survival (OS) time of HGG patients with RAB32 overexpression was shorter, and the results from the two databases were consistent **(Figure 3A, D)**. Then, to further verify the prognostic effect of RAB32 on glioma patients, receiver operating characteristic (ROC) curves were generated based on two databases, and the diagnostic value of RAB32 in glioma was evaluated according to the area under the curve (AUC). In the CGGA database, the AUCs for 1-year, 3-year, and 5-year survival were 0.60, 0.67, and 0.69, respectively. In the TGGA database, the AUCs for 1-year, 3-year and 5-year survival were 0.76, 0.80 and 0.83, respectively** (Figure 3B, E)**. In addition, univariate and multivariate Cox regression analyses were performed on the CGGA OS data of glioma patients to explore whether RAB32 is an independent prognostic factor of glioma. Univariate analysis showed that RAB32 (HR = 2851, p < 0.001), age (HR = 1.665, p < 0.001), IDH mutation (HR = 0.336, p < 0.001), 1p19q (HR = 0.237, p < 0.001) and the chemotherapy (HR = 0.775, p = 0.011) was a prognostic factor for OS in glioma patients. Similarly, multivariate analysis showed that RAB32 (HR = 1.735, p < 0.001), age (HR = 1.528, p < 0.001), IDH mutation (HR = 0.610, p < 0.001), and 1p19q were all absent (HR = 0.381, p < 0.001) and were prognostic factors for OS in glioma patients** (Figure 3C, F)**. These findings suggest that RAB32 is associated with poor prognosis in glioma patients.

### Gene Set Enrichment Analysis (GSEA)

**Figure 4:**
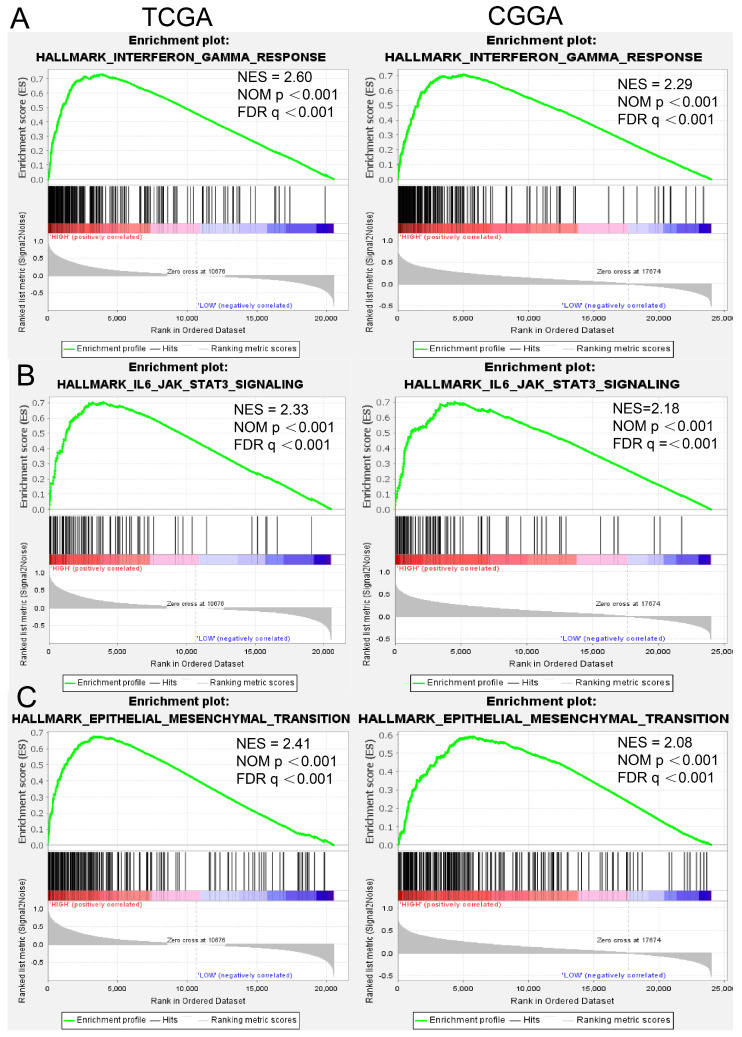
**GSEA** (A) Interferon γ response in TCGA and CGGA. (B) IL6-AKT-STAT3 signaling pathway in TCGA and CGGA. (C) Epithelial mesenchymal transition in TCGA and CGGA.

To gain additional insights into the functional role of RAB32 in gliomas, we performed GSEA using the TCGA and CGGA databases to identify significantly activated pathways in patients with high RAB32 expression in GBM as compared to those with low RAB32 expression. Our results revealed that 15 pathways, including the interferon gamma response, IL-6/JAK/STAT3 signaling pathway, and Epithelial-mesenchymal Transition (EMT), were significantly activated in GBM patients with RAB32 overexpression (NES > 1.5, FDR < 0.25, and p < 0.001). Detailed results are displayed in Table 1, while Figure 4 illustrates the three most significant pathways in terms of p values.

### DNA hypomethylation is associated with RAB32 mRNA expression and poor prognosis in glioma patients

**Figure 5:**
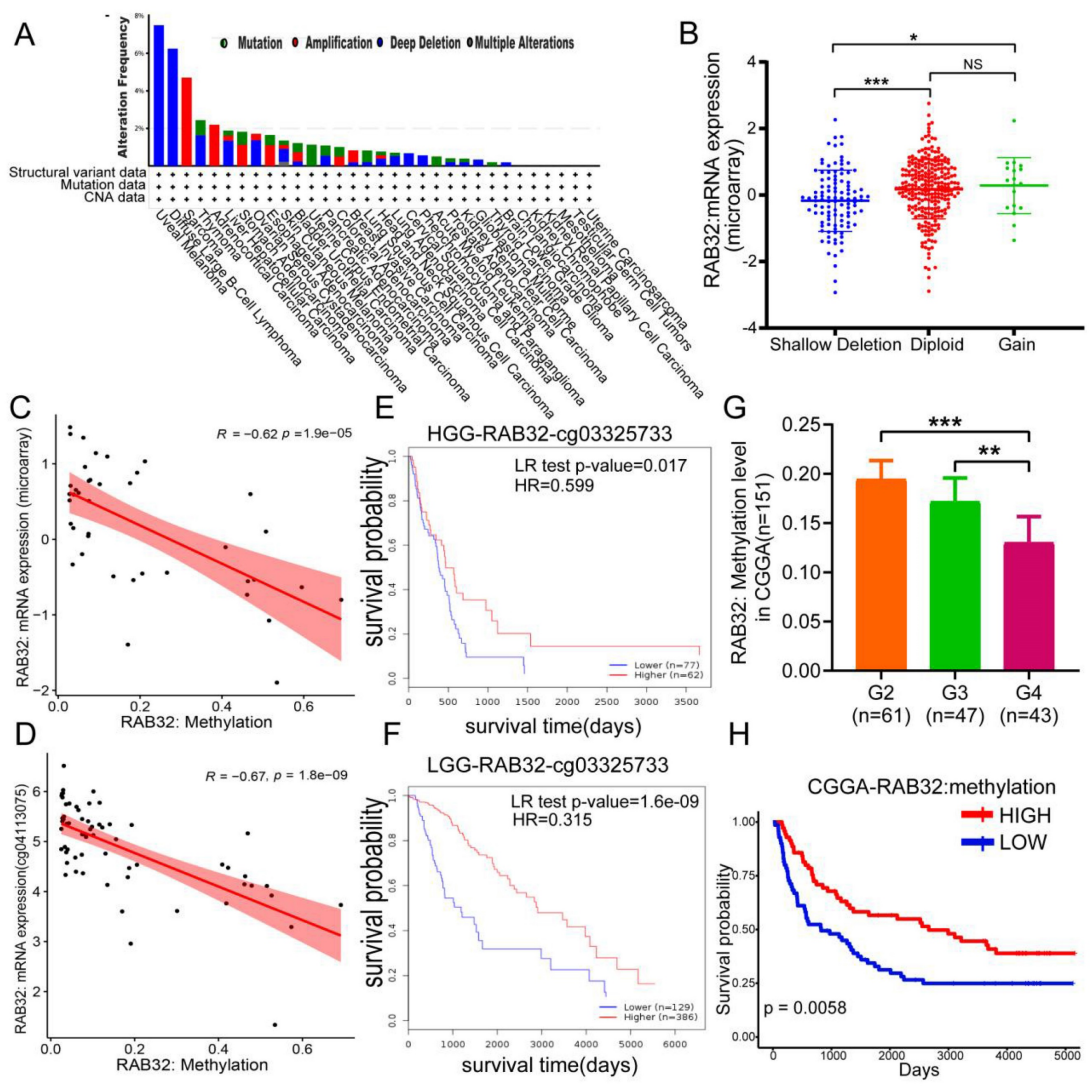
** Analysis of RAB32 gene copy number variation (CNV) and DNA methylation in glioma.** (A) Variation frequency and mutation type of RAB32 in cancer. (B) Changes in RAB32 expression levels under different copy numbers. (C) Correlation between the RAB32 DNA methylation level and expression in the microarray. (D) Correlation between RAB32 DNA methylation level and expression in probe cg04113075. (E) Overall survival curve of RAB32 DNA methylation in high-grade gliomas. (F) Overall survival curve of RAB32 DNA methylation in low-grade gliomas. (G) Correlation between glioma grade and RAB32 DNA methylation level in CGGA data. (H) The overall survival curve based on RAB32 DNA methylation level grouping in CGGA data. *p < 0.05, **p < 0.01, ***p < 0.001. NS, not significant.

After verifying the expression of RAB32, we were interested in the reason for the high expression of RAB32. First, we used the cBioPortal database to study the gene copy number variation of RAB32, observed the genetic changes in RAB32 in all different tumor samples of TCGA, and found that the "deep deletion" type was the main type of cancer, including GBM (**Figure 5A**). Then, we explored the association between RAB32 mRNA expression and its copy number variation from this database. The data showed that only 18 patients had an increased copy number of RAB32 (**Figure 5B**), so we speculated that copy number variation was not the main factor affecting the high expression of RAB32. We hypothesized that DNA methylation affected the expression of RAB32 and found that the degree of DNA methylation was negatively correlated with mRNA (**Figure 5C**). In support of our conclusions, we found a significant negative correlation between RAB32 DNA methylation and the expression of RAB32 mRNA and its subtypes of probes in the nonpromoter region, such as cg04113075 (**Figure 5D**), from the SMART online analysis platform (Figure S2), Afterward, we became very interested in whether the DNA methylation level of RAB32 affected the prognosis of glioma patients. To address this, we used the MethSurv database to explore RAB32 DNA methylation in patients and found that among both HGG patients (**Figure 5E**) and LGG patients (**Figure 5F**), those with a low RAB32 DNA methylation degree had a worse OS than those with a high DNA methylation degree. To verify the influence of the RAB32 DNA methylation level on glioma, we downloaded the methylation data of glioma patients from the CGGA database and found that the WHO grade of glioma was negatively correlated with the methylation level of RAB32 (**Figure 5G**), that is, the higher the expression level of RAB32 was, the lower the methylation level would be. Survival analysis using Kaplan‒Meier analysis also demonstrated that patients with higher RAB32 methylation levels had a better OS (**Figure 5H**).

### RAB32 expression levels were highly correlated with immune cell infiltration in the tumor microenvironment

**Figure 6:**
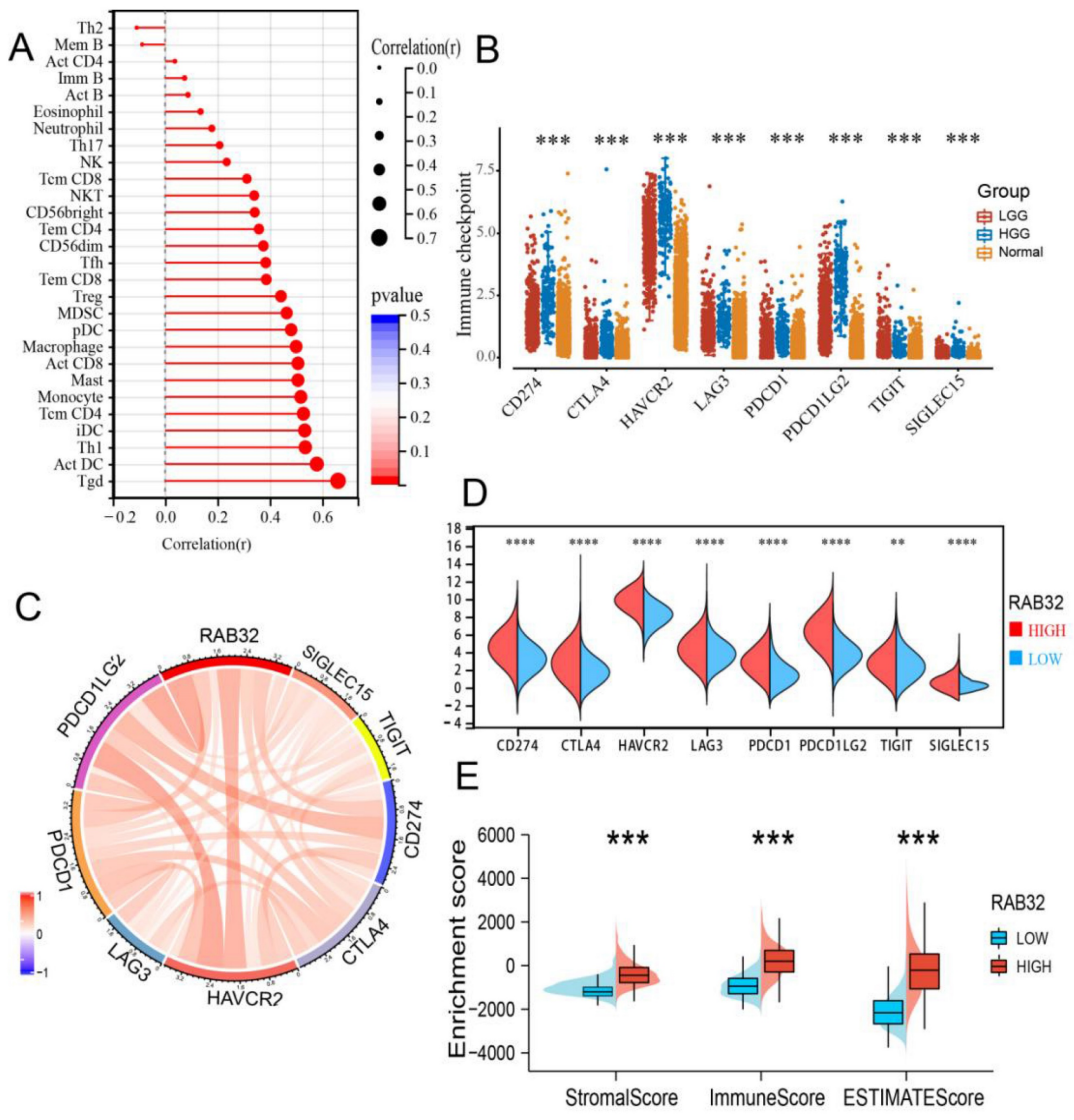
** Correlation between the RAB32 expression level and infiltration of the tumor microenvironment.** (A) Correlation between RAB32 mRNA expression and tumor-infiltrating lymphocytes. (B) Expression of immune checkpoint molecules in gliomas. (C) Correlation between RAB32 and immune checkpoint molecules in glioma. (D) Differential expression of immune checkpoint molecules in groups with high or low RAB32 expression. (E) Correlation between RAB32 mRNA expression and matrix score, immune score and comprehensive score. *p < 0.05, **p < 0.01, ***p < 0.001, ****p < 0.0001.

Using the TISIDB database, we investigated the correlation between RAB32 mRNA expression and tumor-infiltrating lymphocytes (TILs). Our findings indicate that almost all TILs exhibited a positive correlation with RAB32 expression **(Figure 6A)**. Immune checkpoint molecules also play a critical role in immune regulation and autoimmune tolerance. Compared to normal brain tissue, the expression of all immune checkpoint molecules was observed to be upregulated in gliomas, with a more significant upregulation noted in high-grade gliomas (HGG)** (Figure 6B)**. Dividing RAB32 into high- and low-expression categories based on its median expression level, we further explored the correlation between RAB32 mRNA expression and immune checkpoint molecules. Our results revealed a significant correlation between RAB32 and all immune checkpoint markers **(Figure 6C)**. Moreover, glioma patients with high RAB32 expression exhibited higher expressions of immune checkpoint molecules than those with low RAB32 expression **(Figure 6D)**. Using the ESTIMATE algorithm score to predict tumor purity, we found that tumor tissues with high RAB32 expression harbored more stromal and immune cells than those with low RAB32 expression, with the ESTIMATE score also being higher **(Figure 6E)**.

### Knockout of RAB32 inhibited the proliferation, migration and invasion of glioma cells

**Figure 7:**
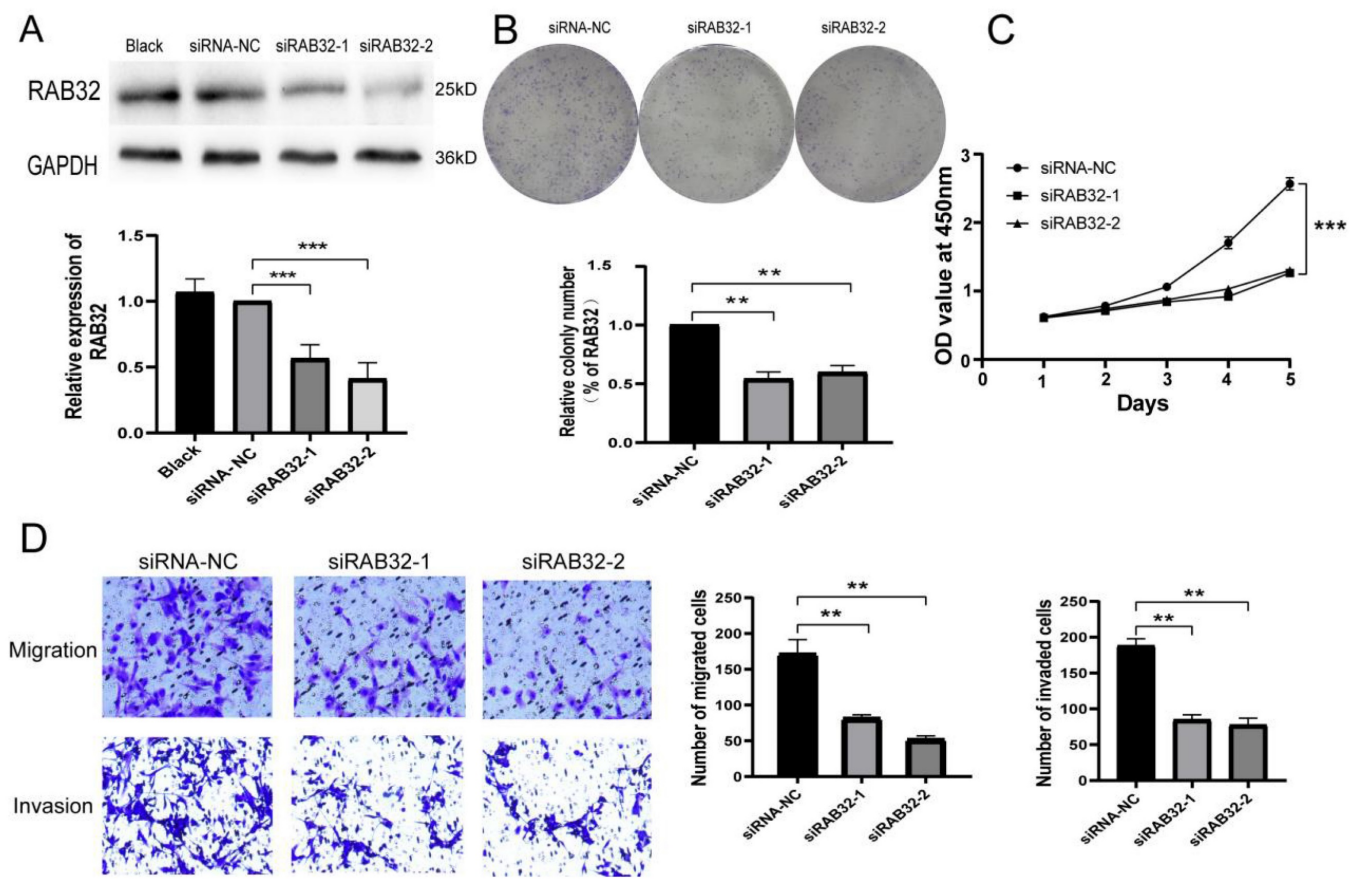
** RAB32 knockout inhibited the proliferation, migration and** invasion of glioma cells. (A) The expression of RAB32 mRNA in transfected U87 cells was detected by western blot. (B, C) Colony formation assay and CCK-8 assay showed that RAB32 knockdown inhibited the proliferation of glioma cells. (D) RAB32 knockdown inhibited glioma cell migration and invasion. Blue is the colony of glioma cells(7B)and glioma cells(7D). *p < 0.05, **p < 0.01, ***p < 0.001.

Previous experiments established that glioma U87 cells exhibited relatively high expression levels of RAB32. To assess the impact of RAB32 expression knockdown, we transfected U87 cells with lentivirus carrying RAB32 siRNA. We evaluated the transfection efficiency by detecting GFP fluorescence via fluorescence inverted microscopy, revealing that more than 90% of the cells in each group exhibited fluorescence **(Figure S3)**. Western blot analysis confirmed that there were no significant differences in RAB32 expression levels between the blank and siRNA-NC groups (p > 0.05), indicating that the lentivirus transfection did not affect the expression of RAB32. Conversely, transfection with RAB32 siRNA resulted in significantly decreased expression levels of the RAB32 gene in U87 cells in the siRAB32-1 and siRAB32-2 groups compared to the siRNA-NC group **(Figure 7A)**, signifying successful transfection and RAB32 expression downregulation. Colony formation assays and CCK-8 assays revealed that RAB32 knockdown significantly impeded U87 cell proliferative ability** (Figure 7B, C)**. Lastly, Transwell assays illustrated that RAB32 deletion reduced the migratory and invasive ability of U87 cells** (Figure 7D)**.

## Discussion

RAB32 has emerged as an oncogene playing a pivotal role in the pathogenesis of various malignancies. Multiple studies have indicated that this protein holds promise as a valuable diagnostic and prognostic biomarker for hepatocellular carcinoma (HCC). Elevated expression of RAB32 has been associated with enhanced tumor cell proliferation and invasion, while higher levels of RAB32 have been statistically correlated with poorer patient outcomes in HCC^19^. Moreover, there is evidence implicating RAB32 in the accelerated progression of ovarian cancer (OV), potentially leading to reduced patient survival times^20^. Conversely, in the context of hematological malignancies, increased expression of the RAB32 gene has been observed to suppress cancer cell proliferation, invasion, and migration while promoting apoptosis and inducing cell cycle arrest^21^. These findings collectively unravel the multifaceted biological role of RAB32 in cancer progression and underscore its potential as a target for anticancer therapies across various tumor types. Nevertheless, further investigation is warranted to fully elucidate the precise role of RAB32 in GBM, thereby clarifying its implications for glioma diagnosis and treatment.

Our investigation of glioma cell and tissue microarrays reveals a significant upregulation of Rab32 in glioma specimens, which demonstrates a positive correlation with tumor grade and unfavorable patient prognosis. Suppression of Rab32 expression leads to a corresponding decrease in the proliferative, migratory, and invasive capabilities of GBM cells. These findings suggest that Rab32 may emerge as a novel molecular marker for enhancing prognostic assessment and improving the quality of life for glioma patients.

This investigation has established the potential of RAB32 as a diagnostic indicator for glioma prognosis, while also indirectly elucidating its influence on cell signaling pathways through gene set enrichment analysis (GSEA). However, the underlying mechanisms regulating RAB32 and its specific effects on GBM remain incompletely understood. Further experimental validation, coupled with more rigorous computational models, is imperative to confirm the role of RAB32 in GBM regulation and provide insights for the development of more rational control strategies. Additionally, accumulating evidence suggests that RAB32 interacts with cellular mitochondria to modulate mitochondrial function and influence cell apoptosis^9^. Therefore, integrating flow cytometry and other experimental methods with sophisticated data analysis models could enhance the study of RAB32-mediated apoptosis in GBM cells, offering a comprehensive understanding of the biological functions of RAB32 in GBM.

A growing body of research suggests that DNA methylation, a primary epigenetic modification, plays a pivotal role in the pathogenesis of various tumors. Moreover, aberrant promoter methylation has emerged as a promising biomarker for diagnosing and prognosticating gliomas^22^. In this study, we aimed to elucidate the potential mechanism underlying the upregulation of RAB32 by considering both genetic and epigenetic alterations. Our findings revealed a significant positive correlation between an increase in RAB32 copy number and its upregulation, as well as a notable inverse relationship between RAB32 mRNA expression and its methylation level. Additionally, we observed substantially lower methylation levels in the RAB32 gene promoter region among GBM patients compared to those with LGG, highlighting a potential association with poorer prognosis. Recent studies have identified a hypomethylated region upstream of RAB32. And its expression is negatively correlated with the methylation of this region^23^. Additionally, it is noteworthy that CpG_24 in RAB32 overlaps with a putative c-Jun binding site, which has been extensively reported as a methylation-sensitive transcription factor^24^,^25^. Our bioinformatics analysis revealed a negative correlation between RAB32 expression and DNA methylation in gliomas. Therefore, elucidating the mechanism of RAB32 DNA methylation and its role in GBM development may provide a scientific basis for the development of novel therapeutic strategies targeting RAB32 or its associated signaling pathways.

Our analysis revealed a positive correlation between the expression of RAB32 and the infiltration of tumor-infiltrating lymphocytes, suggesting that gliomas with high levels of RAB32 expression harbor an increased abundance of stromal and immune infiltrating cells, consequently leading to a decrease in tumor purity. Previous studies have demonstrated that as malignancy progresses, higher-grade gliomas exhibit elevated stromal and immune scores while experiencing a decline in tumor purity. This reduction in tumor purity has been associated with unfavorable prognostic outcomes in patients with GBM. Cancer cells often employ strategies to evade immune surveillance, including the promotion of immune tolerance and evasion of immune destruction. Our data revealed a significant correlation between the expression levels of immune checkpoint molecules (CD274, CTLA4, HAVCR2, LAG3, PDCD1, PDCD1LG2, TIGIT, and SIGLEC15) and RAB32 in high-grade gliomas (HGG). High levels of these molecules were observed in HGG tumors. The upregulation of these immune checkpoint molecules can suppress T-cell-mediated immune responses in glioblastoma multiforme (GBM), facilitating the process of evading the host's immune system^26^. Building upon the success achieved by immunotherapy targeting immune checkpoints in various cancers^27^,^28^, including promising results from clinical trials, our findings suggest that modulating the tumor microenvironment and targeting GBM through inhibition of RAB32 could be a potential therapeutic strategy.

In conclusion, our findings indicate a significant upregulation of RAB32 in GBM cell lines and tumor tissues, which enhances the proliferation and invasion capabilities of GBM. Moreover, it serves as a prognostic indicator for poor prognosis. Additionally, RAB32 plays a crucial role in tumor immunity by influencing the malignant progression of glioma and patient survival. Further experiments should be conducted to validate the correlation between RAB32 expression and immune infiltration, as well as to investigate whether immunotherapy can effectively inhibit RAB32 expression to improve the prognosis of glioma patients. Therefore, RAB32 holds promise as both a biomarker and potential therapeutic target for glioma treatment.

## Supplementary Material

Supplementary figures.

## Figures and Tables

**Table 1:** RAB32 signaling pathway in glioma based on two datasets

Pathways	TCGA RNA-seq				CGGA RNA-seq		
	NES	NOM p value	FDR q-value		NES	NOM p value	FDR q-value
Interferon γ response	2.60	< 0.001	< 0.001		2.29	< 0.001	< 0.001
Inflammatory response	2.50	< 0.001	< 0.001		1.92	< 0.001	< 0.001
Interferon α response	2.43	< 0.001	< 0.001		2.28	< 0.001	< 0.001
TNFa Signaling via NF-KB	2.41	< 0.001	< 0.001		2.08	< 0.001	< 0.001
EMT	2.41	< 0.001	< 0.001		2.08	< 0.001	< 0.001
IL6/JAK/STAT3 Signaling	2.33	< 0.001	< 0.001		1.91	< 0.001	< 0.001
IL2/STAT5 Signaling	2.16	< 0.001	< 0.001		1.85	< 0.001	< 0.001
Coagulation	2.16	< 0.001	< 0.001		1.88	< 0.001	< 0.001
Complement	2.14	< 0.001	< 0.001		1.98	< 0.001	< 0.001
E2F Targets	2.08	< 0.001	< 0.001		1.68	< 0.001	< 0.001
Apoptosis	2.08	< 0.001	< 0.001		1.89	< 0.001	< 0.001
Hypoxia	1.86	< 0.001	< 0.001		1.85	< 0.001	< 0.001
Glycolysis	1.70	< 0.001	0.001		1.74	< 0.001	< 0.001
P53 Pathway	1.62	< 0.001	0.002		1.76	< 0.001	< 0.001
MTORC1 Signaling	1.56	< 0.001	0.006		1.83	< 0.001	< 0.001

NES normalized enrichment score, NOM nominal, FDR false discovery rate, EMT epithelial mesenchymal transition. Gene sets with NOM P value<0.05 and FDR q-value<0.25 were considered significantly enriched.
