# Supplementary material for: High Expression of RAB32 Predicts Adverse Outcomes: A Potential Therapeutic Target for Glioblastoma
Source: J Cancer. 2024 Oct 28;15(20):6710–23. doi: 10.7150/jca.96162 (PMC11632980; doi:10.7150/jca.96162)
Supplement: Supplementary file 1 — Supplementary figures. [file jcav15p6710s1.pdf]

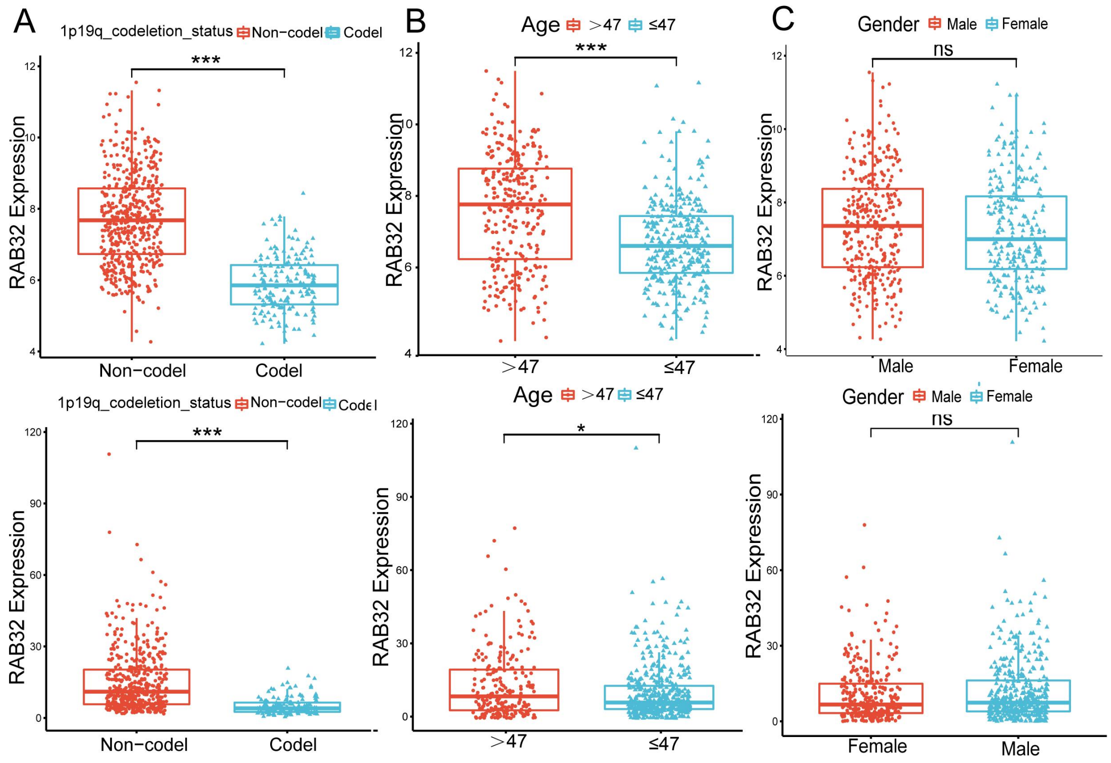

Supplement Figure 1

(A) Correlation between RAB32 mRNA and 1p19q chromosome deletion in glioma patients. (B) Correlation between RAB32 mRNA and age of glioma patients. (C) Correlation between RAB32 mRNA and gender of glioma patients. The data above is from TCGA, and the data below is from CGGA. *p<0.05, **p<0.01, ***p<0.001, ns not significance.

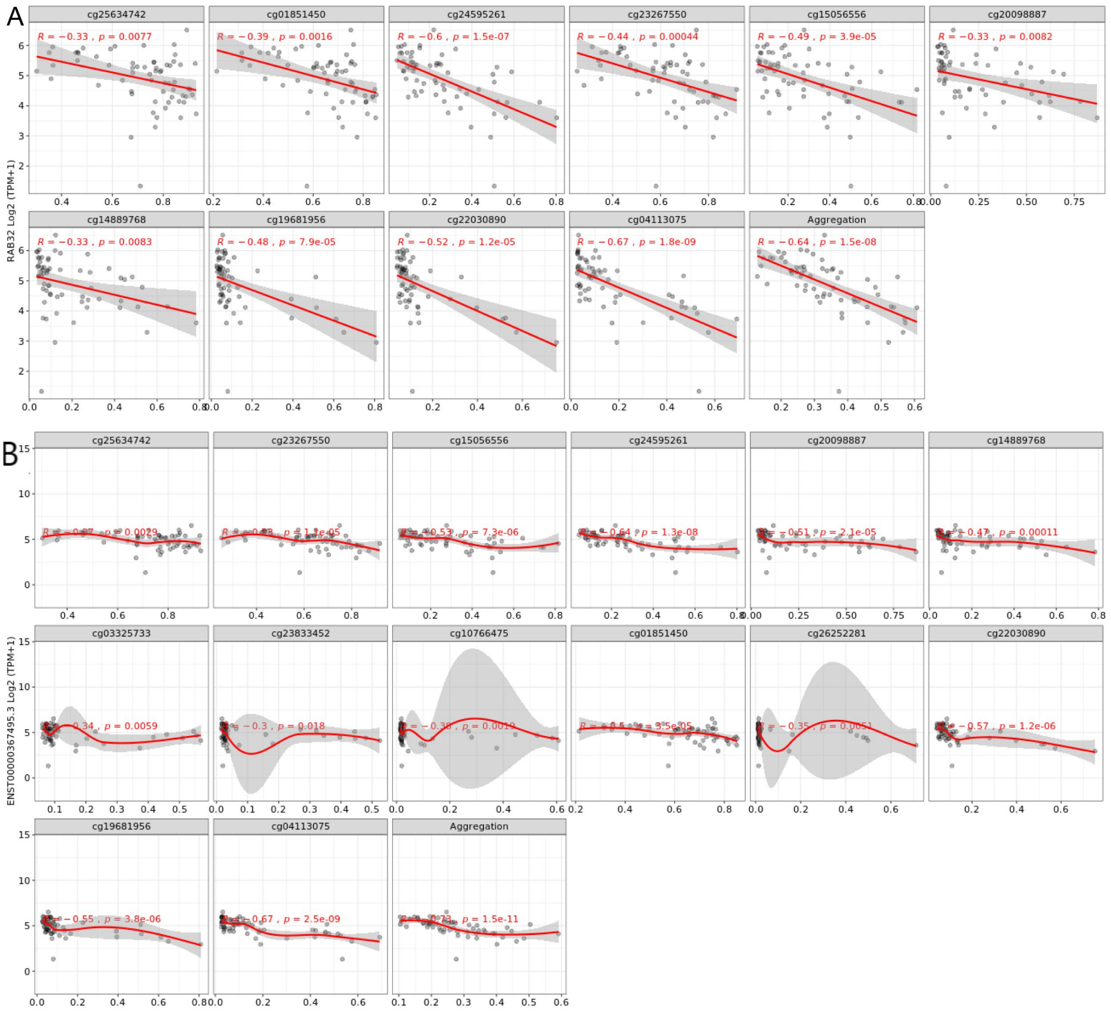

Supplement Figure 2

(A) Correlation between RAB32 mRNA expression and methylation level. (B) Correlation between RAB32 subtype mRNA expression and methylation level. The horizontal axis represents the level of methylation, and the vertical axis represents the level of mRNA expression.

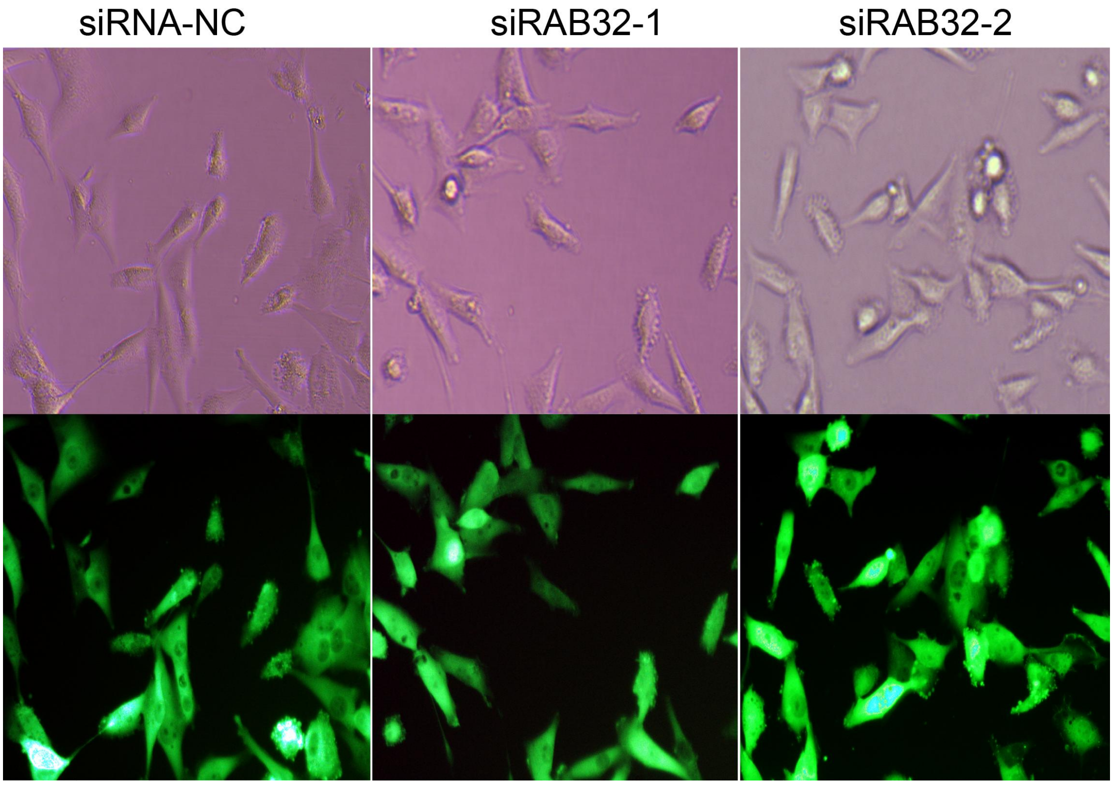

Supplementary Figure 3

Fluorescence expression of GFP in U87 cells after transfection with lentivirus
